# Avoidance of Radiotherapy-related, Gastrointestinal Complications in a Patient with Systemic Lupus Erythematosus: A Case Report and Review of Literature

**DOI:** 10.4103/1319-3767.56103

**Published:** 2009-10

**Authors:** Eyad Alsaeed

**Affiliations:** Division of Radiation Oncology, King Fahad Medical City, Riyadh, Saudi Arabia

**Keywords:** Gastrointestinal tract side effects, intensity-modulated radiotherapy technique, systemic lupus erythematosus

## Abstract

Systemic lupus erythematosus (SLE) is associated with major gastrointestinal complications due to radiotherapy. A patient with active SLE and grade 4 nephropathy presented with inoperable advanced cancer of the cervix which proved to be contraindicated for chemotherapy. The patient was treated with intensity-modulated radiotherapy technique (IMRT). The patient, however, did not experience severe radiotherapy-related complications as expected with conventional techniques of radiotherapy. The tolerance of SLE patients to radiotherapy can thus be achieved by proper delivery of radiation and the sparing of normal tissues by IMRT although further confirmatory studies are required.

Severe gastrointestinal tract (GIT) complications induced by radiotherapy (RT) have been well documented in patients with systemic lupus erythematosus (SLE). SLE is a chronic, inflammatory, multi-system disease characterized by remissions and exacerbations. Its pathogenesis shows multiple, immunological aberrations characterized by polyclonal B-cell activation associated with abnormal expression of cytokines.[1,2]

The use of RT in SLE patients has been considered to be a relative contraindication by many oncologists due to these patients' intolerance to radiation-induced side effects.[3–5] In SLE patients, RT induces inflammatory reactions to the irradiated tissues, which lead to exacerbation of the inflammatory process. These radiation-induced side effects are related to the dose of RT and the volume of the irradiated normal tissue, thus, the goal of RT is to deliver an optimal dose to the tumor, with a minimal irradiated volume of normal tissues, including the GIT.[3]

Intensity-modulated radiotherapy (IMRT) is a new radiotherapy technique that uses an inverse planning algorithm and allows radiation oncologists to be able to decide the exact dose of radiation to be delivered to the tumor and normal tissues.[6]

## CASE REPORT

A 32 year-old lady known to have SLE for eight years, and suffering from hypertension and grade IV lupus nephritis (proved by renal biopsy), presented with irregular vaginal bleeding and dysparaunia. Pelvic examination showed a bulky endocervical mass extending to the parametrium, but not reaching the pelvic wall, with an upper third vaginal wall extension. Biopsy revealed a high-grade squamous cell carcinoma. Thoracic, abdominal, and pelvic computed tomography (CT) and magnetic resonance imaging (MRI) confirmed the extension of the tumor to the lower uterine segment, which was associated with enlargement of the internal iliac pelvic lymph nodes without any evidence of metastasis. In addition, a bone scan was performed and showed no bony metastasis. Based on this work-up, the patient was diagnosed with stage IIB cervical cancer.

Her renal function was normal as proved by a renogram. Antinuclear antibody titer was 320, antiDNA antibody titer was 361, and other immunological study results were normal. Chemotherapy was contraindicated due to lupus nephropathy and surgery was not performed due to the extension and size of the tumor. Based on the contraindication to have neither surgery nor chemotherapy, the decision was made to treat her with RT. She received local 45 Gray (Gy) pelvic irradiation in 25 fractions (fx) over five weeks, 1.8 Gy/fx, with the IMRT technique. The radiation dose to the bowel was calculated to be as low as possible (see below).

**Figure 1:**
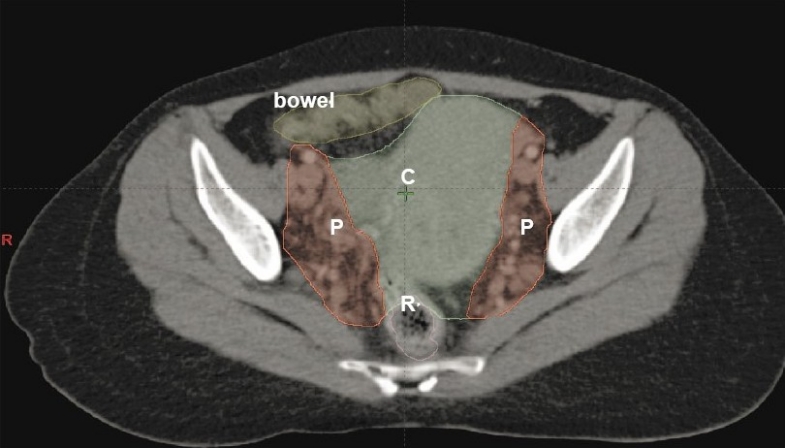
Delineated cervix and uterus in color (C), both parametria and LNs in red colour (P), rectum in orange color and bowel in yellow color

**Figure 2:**
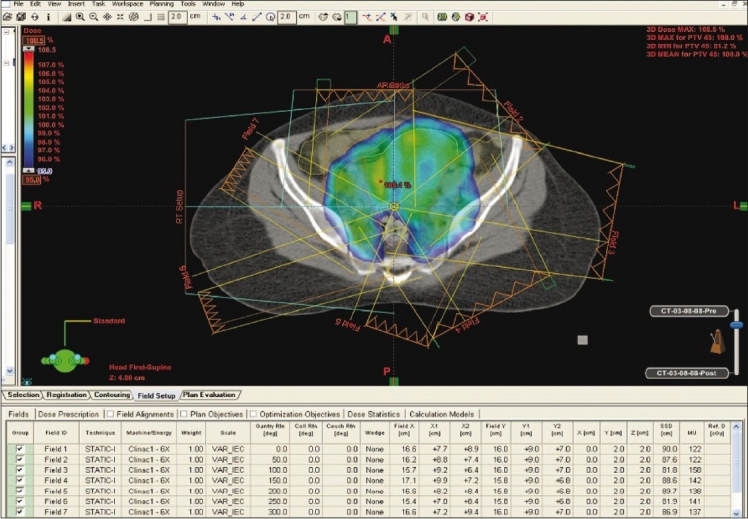
Isodose curve in color wash showing optimal dose shaping to the target volume with sparing of the rectum and bowel

**Figure 3:**
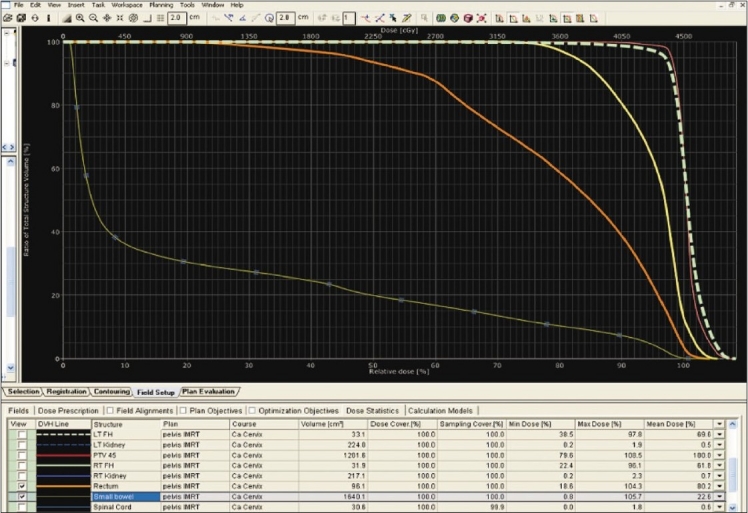
Dose-volume histogram showing the dose to the normal tissues and the clinical target volume

The target volume and bowel were delineated with the aid of CT with contrast and fused MRI images as shown in Figure 1. The doses to the target and normal tissues were optimized as shown in Figure 2. The dose-volume histogram (DVH) showed a delivery of a 100% mean dose to the planning target volume (minimal dose 90% and maximal dose 108.5% as shown in Figure 3). The dose to the rectum ranges from 18.6 to 104.3% with a mean dose of 80%. The differential dose was V_45Gy_ (the rectal volume receiving > 45 Gy) < 1%, V_40 Gy_ of 40%, and V_30 Gy_ of 77%.

It is evident from the DVH that the small bowel dose ranges from 1 to 105.7% with a mean dose of 22.6%. The differential doses were V_45 Gy_ = 0.4%, V_40 Gy_ = 7.6%, and V_20Gy_ = 22%.

### Acute GIT toxicity

The acute GIT side effects were graded and recorded using the common toxicity criteria modified by the National Cancer Institute of Canada (CTC-NCIC).[7] The gastrointestinal symptoms experienced were: i) nausea which was expected even in nonSLE patients in week 1 and that had disappeared at the end of the 1^st^ week, ii) diarrhea which is the most worrisome expected side effect that started in the fourth week (after administering 36 Gy) and that was not more than four motions/day over the baseline (once daily). The rectal discomfort during defecation (G1 proctitis) started to appear in week 5 as shown in Table 1.

**Table 1:** Gastrointestinal toxicity

GIT toxicity	W1	W2	W3	W4	W5
Nausea	G1	G0	G0	G0	G0
Diarrhea	G0	G0	G0	G1	G1
Proctitis	G0	G0	G0	G0	G1

W: week

## DISCUSSION

It is challenging to use radiotherapy in SLE patients due to severe acute and late side effects.[8] In this case, the aim was to reduce the dose of radiation therapy to normal tissues and to give the prescribed dose to the tumor by using the IMRT technique, although there is limited experienced in such cases. Most of the previously published studies showed that conventional radiotherapy induces acute and late GIT complications in SLE patients[8–10] with limited experience with the IMRT technique.[6]

In their review of the Michigan University experience, Lin and his colleagues showed that 86 patients had collagen vascular diseases and concluded that radiotherapy is associated with moderate side effects, but they were nevertheless not prohibitive for its use.[9] Furthermore, these results coincide with the Mayo Clinic data on 22 SLE patients thatshowed that whereas radiotherapy was well tolerated by these patients, it did carry a high risk of severe, late toxicity.[10]

This report reveals that decreasing the radiation dose to the normal tissues by using IMRT is associated with mild side effects. However, more data are needed to reach conclusions in the use of radiation therapy in patients with active connective tissue disease, including SLE.
